# Phasic Diastolic Coronary Artery Compression: A Rare Cause of Chest Pain at Rest

**DOI:** 10.7759/cureus.38883

**Published:** 2023-05-11

**Authors:** John A Gambril, Gbemiga Sofowora, Umair Ahmad

**Affiliations:** 1 Pediatrics, Nationwide Children’s Hospital, Columbus, USA; 2 Internal Medicine, The Ohio State University Wexner Medical Center, Columbus, USA; 3 Cardiovascular Medicine, The Ohio State University Wexner Medical Center, Columbus, USA

**Keywords:** coronary artery angiogram, phasic diastolic coronary artery compression, radiation cardiotoxicity, pericardial adhesion, chest pain at rest

## Abstract

Phasic diastolic coronary artery compression (PDCAC) is a rare phenomenon caused by the compression of a coronary artery between expanding myocardium and a non-compliant overlying structure. We report a unique case of an elderly female who presented with recurrent paradoxical substernal chest pain at rest caused by PDCAC of the proximal left circumflex artery (LCx). Her chest pain likely occurred at rest due to longer diastolic compression time at slower heart rates. Pericardial adhesion secondary to past breast radiation was the likely cause of PDCAC. She was treated successfully with oral anti-hypertensive and anti-anginal medical therapy. PDCAC is a rare phenomenon but should be on the differential for chest pain occurring at rest, especially if there is a history of mediastinal or cardiac radiation or inflammation. PDCAC treatment depends on the underlying cause but can be treated successfully with medical therapy alone.

## Introduction

Phasic coronary artery compression is most commonly seen in systole, usually caused by congenital myocardial bridging. Phasic diastolic coronary artery compression (PDCAC), on the other hand, is a rare, acquired phenomenon. It is caused by compression of an epicardial coronary artery between expanding myocardium and an overlying non-compliant structure [1]. Various etiologies of these non-compliant overlying structures have been reported [1-5]. Presentations and treatment are, not surprisingly, variable case-to-case depending on the underlying cause [1,3,6]. Here, we report a case of a 79-year-old female who presented with recurrent substernal chest pain that occurred only at rest. After extensive workup, she was found to have PDCAC of the left circumflex artery (LCx). We discuss the likely etiology of PDCAC in our patient, propose a mechanism for the presentation of chest pain at rest, discuss management considerations, and review the limited literature on PDCAC.

## Case presentation

**Figure 1:**
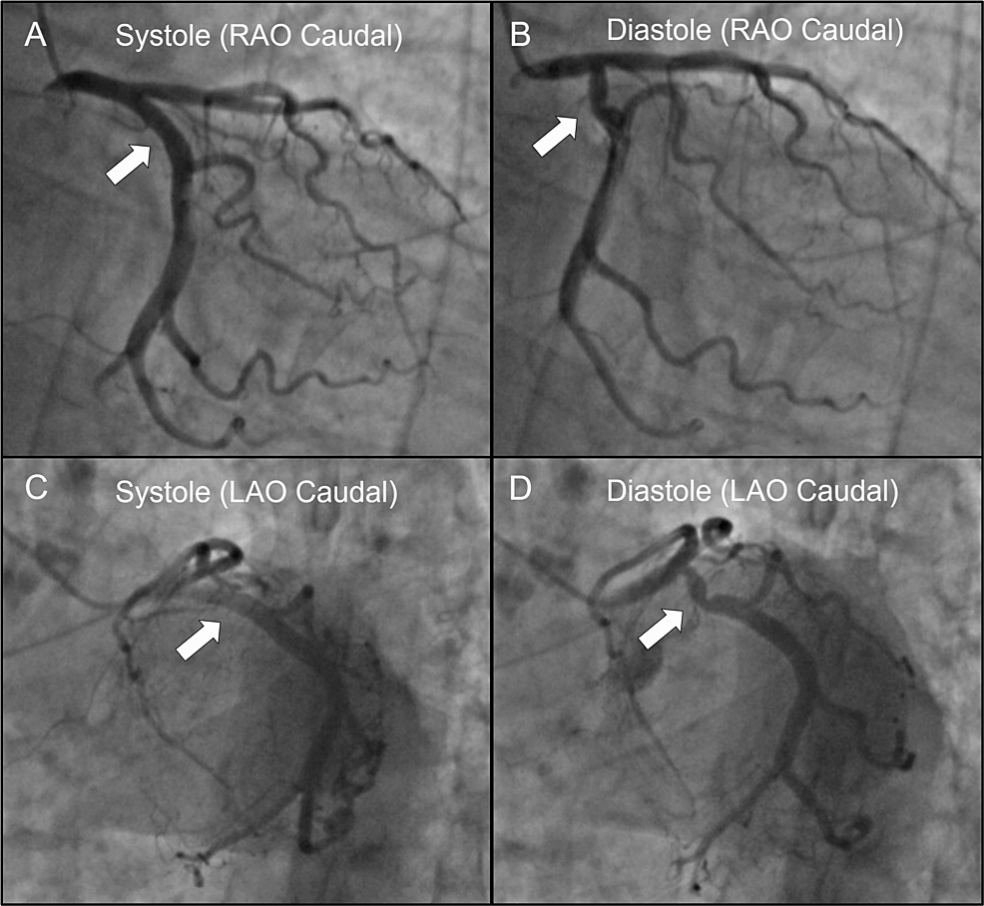
Phasic diastolic compression of the proximal left circumflex artery (LCx). Right anterior oblique (RAO) caudal view seen in systole (A) and diastole (B). Left anterior oblique (LAO) caudal view seen in systole (C) and diastole (D). Arrows indicate the location of phasic diastolic compression of the LCx. In systole (A, C) the LCx is widely patent. In diastole (B, D) the LCx displays dramatic, localized kinking.

A 79-year-old female with hypertension and a remote history of right-sided breast cancer status post-lumpectomy and radiation therapy presented to the emergency department three times over two months for recurrent substernal chest pain. She described the symptoms as both substernal chest pain and pressure that felt "as if someone was sitting on my chest." There was associated diaphoresis but no syncope, palpitations, shortness of breath, visual changes, or weakness. Symptom onset occurred only at rest, multiple times waking her from sleep. She never experienced symptoms with exertion. Initial vitals for each of her visits were notable for elevated blood pressure, around 150 mmHg systolic, but otherwise stable within normal limits. Repeated physical examinations were unrevealing. Cardiac examination showed normal heart rate and regular rhythm without murmurs, normal S1 and S2 without S3 or S4, strong and equal peripheral pulses, no pericardial rub, no jugular venous distention, and no peripheral edema. Pulmonary examination showed good bilateral air movement without adventitious breath sounds or chest wall deformity. Repeated electrocardiograms showed her baseline tracing of normal sinus rhythm with no conduction abnormalities, ST changes, or Q waves. Labwork was unrevealing. The complete metabolic panel, complete blood count, lipase, and serial troponins were within normal limits each time. CT pulmonary angiography revealed no pulmonary embolism. Transthoracic echocardiography showed normal size of all chambers, normal systolic and diastolic function of all chambers, no wall motion abnormalities, left ventricular ejection fraction of 60-65%, no significant valvular disease, and no pericardial effusion. The pharmacological nuclear stress test showed no evidence of ischemia. A left heart catheterization was finally obtained for coronary angiography. This revealed a left dominant system with no identifiable luminal disease in any vessel. However, there was dramatic phasic diastolic compression of the proximal LCx (Figure 1, Videos 1, 2). No percutaneous coronary intervention was performed at this time, in favor of a conservative medical approach. The patient was sequentially started on daily amlodipine, twice daily carvedilol, daily isosorbide mononitrate, and as-needed sublingual nitroglycerin as both anti-hypertensive and anti-anginal therapy. As of this writing, she is 18 months removed from diagnosis and initiation of medical therapy. She has experienced just two additional episodes of chest pain (both at rest), and her blood pressure at follow-up visits has been at goal.

**Video 1:** Coronary Angiography in Right Anterior Oblique Caudal View - Phasic diastolic compression of the proximal left circumflex artery (LCx) is demonstrated. During systole, the LCx is widely patent. But as the heart expands during diastole, the proximal LCx can be seen to dramatically kink. This repeats with every cardiac cycle.

**Video 2:** Coronary Angiography in the Left Anterior Oblique Caudal View - Phasic diastolic compression of the proximal left circumflex artery (LCx) is demonstrated. During systole, the LCx is widely patent. During diastole, the LCx dramatically kinks. Systole and diastole can be differentiated in this view by looking at the spacing between the other coronary arteries. During systole (as the heart contracts), they appear to bunch together. During diastole (as the heart expands), they seem to spread out.

## Discussion

Here, we reported the case of a 79-year-old female with PDCAC of the LCx presenting, paradoxically, as substernal chest pain at rest. She was treated successfully with anti-hypertensive and anti-anginal medical therapy.

Though available literature is sparse and limited to case reports, PDCAC of the LCx [5-8], obtuse marginal [7], left anterior descending artery (LAD) [2-4,8], diagonal branches [1,9], and bypass grafts have been reported [10,11]. In the literature, etiologies include local post-operative [1,3,5,10,11] or inflammatory adhesions [2], constrictive pericarditis [3,5,7,8,11], and even ribs [4]. Dilated cardiac chambers and anatomic abnormalities have also been shown to contribute [2,4,6].

**Figure 2:**
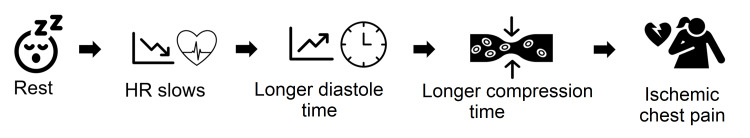
Proposed mechanism for chest pain at rest due to phasic diastolic coronary artery compression.

Other reported presentations of PDCAC include those of congestive heart failure or constrictive pericarditis [2,4-8,10], exertional [3] or unspecified chest pain [2,5,11], or no symptoms at all [1,3,9]. To our knowledge, this is the first reported case of PDCAC presenting specifically as chest pain at rest. Our patient’s presentation can be explained by the fact that coronary perfusion occurs primarily during diastole. When her heart rate is at its slowest at rest and during sleep, her diastole time, and thus coronary compression time, is correspondingly at its greatest. This leads to chest pain paradoxically manifesting at rest (Figure 2). With this in mind, PDCAC should be included in the differential diagnosis for patients with chest pain at rest, especially if there is a history of cardiac or mediastinal inflammation, thus predisposing to pericardial adhesion formation.

So, what caused our patient’s PDCAC? Echocardiography ruled out contribution from dilated cardiac chambers, and she has no history of left atrial appendage devices, a known cause of non-phasic compression of the proximal LCx due to close anatomic relationship [12,13].

In parallel with the existing literature, it is most likely that local adhesions were the culprit of our patient’s PDCAC, with radiation exposure as the most likely cause. We know she had right-sided breast cancer treated with radiation therapy about 10 years prior to presentation. Though more common with left-sided breast cancer, pericarditis is still a known manifestation of radiotherapy-induced cardiotoxicity and can occur with right-sided radiation [14]. Chronic pericarditis is often asymptomatic or subclinical without any overt episode of acute pericarditis. With no other known or suspected pericardial processes or anatomic abnormalities, pericardial adhesions secondary to breast radiation is the most likely cause of PDCAC in our patient.

CT angiography with cardiac function or cardiac magnetic resonance imaging could shed more light on the anatomic relationships and structures contributing. Direct visualization during surgery or autopsy could also give clues. Regardless, this patient has experienced excellent outcomes with medical therapy alone. By reducing her myocardial work and afterload, her chest pain has significantly improved. Roberto and Agarwal reported similar success using oral anti-hypertensive therapy to treat PDCAC of the LCx, though that case was due to compression from a severely dilated left atrium in the setting of systolic heart failure [6]. Other reported cases have been treated successfully with local adhesion resection [2,3], pericardiectomy [5,7,8,11], stenting [1,3], and coronary artery bypass grafting [3]. Varghese and Sanghvi reported a unique case of PDCAC of the LAD [4]. Severe rheumatic mitral valve disease caused severe left atrial dilation and lateral displacement of the left ventricle, leading to diastolic compression of the LAD against ribs five and six. Mitral valve replacement and left atrial appendage (LAA) ligation led to good outcomes, highlighting the importance of tailoring treatment plans to the underlying cause. Should medical therapy alone become insufficient in our patient, stenting is a viable option. However, this should be delayed as long as possible given the possibility of stent deformation secondary to continued compressive forces [3]. Ultimately, surgical resection of the overlying structure can be curative [8].

## Conclusions

PDCAC is rare and occurs due to pinching of an epicardial coronary artery between expanding myocardium and an overlying non-compliant structure, such as pericardial adhesions. We report the case of an elderly female with PDCAC of the LCx presenting as paradoxical substernal chest pain at rest. Her unique presentation is explained by longer interruption of coronary perfusion during diastole while the heart rate is lower. Her PDCAC was most likely caused by pericardial adhesions secondary to past breast radiotherapy. She was successfully treated with oral anti-hypertensive and anti-anginal therapy alone. Percutaneous coronary intervention and surgery remain as future options if needed. PDCAC should be on the differential diagnosis for substernal chest pain occurring at rest, especially if there is a history of mediastinal or cardiac radiation or inflammation.
